# Somatic Antigens of Tropical Liver Flukes Ameliorate Collagen-Induced Arthritis in Wistar Rats

**DOI:** 10.1371/journal.pone.0126429

**Published:** 2015-05-18

**Authors:** Yasir Akhtar Khan, Sadiq Umar, Syed M. A. Abidi

**Affiliations:** 1 Section of Parasitology, Department of Zoology, Aligarh Muslim University, Aligarh, India; 2 Department of Pharmaceutical Science, Collage of Pharmacy, Washington State University, Spokane, Washinton, United States of America; Queen Mary University of London, UNITED KINGDOM

## Abstract

Parasitic helminths polarize immune response of their vertebrate hosts towards anti-inflammatory Th2 type and therefore it is hypothesized that they may suppress the inflammatory conditions in autoimmune disorders. The present study was undertaken to investigate *in vivo* immunomodulatory and therapeutic potential of somatic antigens (Ag) of liver infecting digenetic trematodes [*Fasciola gigantica* (Fg) and *Gigantocotyle explanatum* (Ge)] in collagen-induced arthritic (CIA) Wistar rats. The CIA rats were administered subcutaneously with different doses (50 μg, 100 μg and 150 μg) of somatic antigens of Fg and Ge, daily for 21 days, the time period required to establish infection in natural host (*Bubalus bubalis*). Thereafter, the control, diseased and treated rats were compared for different parameters viz. hind paw thickness; serum interleukins, IL-4 and IL-10, tumor necrosis factor-α (TNF-α) and interferon-γ (IFN-γ); expression level of matrix metalloproteinases (MMPs) -2, -9, -13 and nitric oxide (NO) in knee joints and patellar morphology. The CIA rats treated with different antigens, Fg-Ag and Ge-Ag, show significant amelioration of the disease by down regulation of serum TNF-α and IFN-γ (p< 0.05) and upregulation of IL-4 and IL-10 cytokines (p< 0.05); inhibition (p< 0.05) of MMPs (-2,-9,-13) and NO in knee joints and improved patellar morphology with decreased synovial hypertrophy and reduced infiltration of ploymorphonuclear cells. The activity of pro as well as active MMPs (-2 and -9) and active MMP-13 in knee joints of CIA rats was very high compared to the control and treatment groups, suggesting the extent of collagen degradation in CIA rats. Interestingly, the highest dose (150 μg) of Ge-Ag almost wiped out MMP-13 expression. The overall findings suggest that the somatic proteins of Ge-Ag appeared to be therapeutically more effective than Fg-Ag, reflecting interspecific molecular differences which could contribute to the ability of these worms to successfully ameliorate the pathology of CIA.

## Introduction

Rheumatoid Arthritis (RA) is a systemic autoimmune disease of unknown etiology [1], affecting about 1% of the world population [2]. It is characterized by progressive damage in bone and cartilage that ultimately leads to disability [3, 4].

In RA, the involvement of CD4^+^ cells and matrix metalloproteinases (MMPs) are the hallmark for disease progression and degradation of cartilage [5, 6]. RA is mediated by a disequilibrium of Th1 subset derived cytokines including TNF-α and IFN-γ [7, 8] whereas the Th2 subset exerts antagonistic effect on Th1 subset by producing the anti-inflammatory cytokines including IL-4 and IL-10 [9, 10]. Such effect could be used as a therapeutic target in RA. The inflammatory cytokines such as IL-1β and TNF-α upregulate the expression of MMPs which degrade extracellular matrix proteins and play a central role in RA [11, 12]. The articular chondrocytes preferentially express MMP-13 (collagenase-3) which primarily cleaves type II collagen as well as targets type I, type III collagen and aggrecans [13, 14]. Further cleavage is achieved by the gelatinases, MMP-2 and MMP-9 which can also digest type I collagen and aggrecans [15, 16]. Therefore, the inhibition of different MMPs could also be one of the most effective therapeutic targets to prevent RA. Many synthetic inhibitors have been used to suppress expression of MMPs [17, 18]. The inhibition of cytokine suppressive binding protein / p38 kinase significantly ameliorates the disease in the CIA models, possibly through suppression of MMPs [19]. Besides, the IL-4 is known to inhibit IL-1 induced expression of MMPs while IL-10 suppresses the MMPs by upregulating the expression of TIMP (Tissue inhibitor of metalloproteinase) [20–24]. The protective role of Th2 cytokines such as IL-4 and IL-10 in arthritis has been shown earlier [25]. It has been reported that IL-10 inhibits paw swelling, pannus formation, suppresses pro-inflammatory cytokines and cartilage degradation in CIA rats [25]. Other key modulators in RA are reactive oxygen species (ROS), reactive nitrogen species (RNS) and nitric oxide (NO) which are triggered by pro-inflammatory cytokines, TNF-α, IFN-γ, IL-1 and IL-2 [26–28] while anti-inflammatory cytokines, IL-4, IL-8, IL-10, IL-13 and TGF-β suppress their synthesis [29–31].

The skewing of pro-inflammatory response towards an anti-inflammatory nature and thereby suppression of MMPs, could be a valid therapeutic strategy in RA and that could be achieved by the use of helminth antigens because it has been revealed that ES62 antigen derived from a filarial nematode suppresses TNF-α and IFN-γ in Collagen-induced arthritis [32]. The liver fluke, *Fasciola hepatica* infection polarizes a strong Th2 response leading to antibody class switching and not only inhibit the Th1 response [33], but also attenuate the experimental autoimmune encephalomyelitis through suppression of Th1 and Th17 response [34]. The Th2 response developed in *Fasciola hepatica* infection remains consistent even in chronic infection [35]. *Fasciola hepatica* derived Excretory / Secretory (E / S) products also induce strong Th2 response [36]. Further, these parasites also suppress Th1 derived immune response during *Bordetella pertusis* and *Mycobacterium bovis* infection and inhibit mast cells to mount Th1 response in mice [36–39].

Almost nothing is known about the immunomodulatory role of the tropical liver fluke, *Fasciola gigantica* and amphistome, *Gigantocotyle explanatum* antigens. Though, in our preliminary studies we found a strong Th2 type response against their antigens. But the present study was undertaken to investigate therapeutic efficacy of liver fluke antigens which modulate the cytokines (IL-4, IL-10, TNF-α and IFN-γ), MMPs (-2, -9 and -13), nitric oxide (NO) and patellar morphology in Wistar rats which were subjected to collagen-induced arthritis.

## Materials and Methods

### Animals

Female Wistar rats, weighing about 200g and 8 week old, were purchased from the Animal Facility at Jamia Hamdard Central University (JHCU), New Delhi, India under the license of the Departmental Ethical Committee of Department of Biochemistry, Aligarh Muslim University, which reviewed and approved all the experimental work involving animals. The experimental animals were randomly divided into 4 groups viz. 1. Vehicle control, 2. Collagen-induced arthritis (n = 5 in each group), 3. *Fasciola gigantica* antigens treated and 4. *Gigantocotyle explanatum* antigens treated. The groups, 3 and 4 were further divided into three subgroups (n = 5 in each subgroup) for three different doses of antigens.

### Extraction of somatic antigens of liver flukes

The adult live flukes of *Fasciola gigantica* and *Gigantocotyle explanatum*, obtained from the infected livers of freshly slaughtered Indian water buffaloes at the local abattoir, were thoroughly washed with Hanks’ saline (HBSS) (Fisher Scientific, U.S.A.) followed by a rinse with 0.1M phosphate buffered saline (PBS) (Fisher Scientific, U.S.A.), pH 7.4, containing 0.5% antibiotic / antimycotic solution (Himedia, India) and then incubated for 1 hour at 37°C to allow regurgitation of the gut contents. Thereafter, worms were homogenized in 0.1M PBS, pH 7.4; centrifuged at 10,000×g for 30 min in cooling centrifuge (Eppendorf, Germany) at 4°C. The cell free supernatants of *Fasciola gigantica* (Fg) and *Gigantocotyle explanatum* (Ge) were separately aliquoted and stored at—80°C for subsequent use as antigens (Ag) for treatment purpose.

### Induction of collagen-induced arthritis and treatment schedule

**Fig 1:**
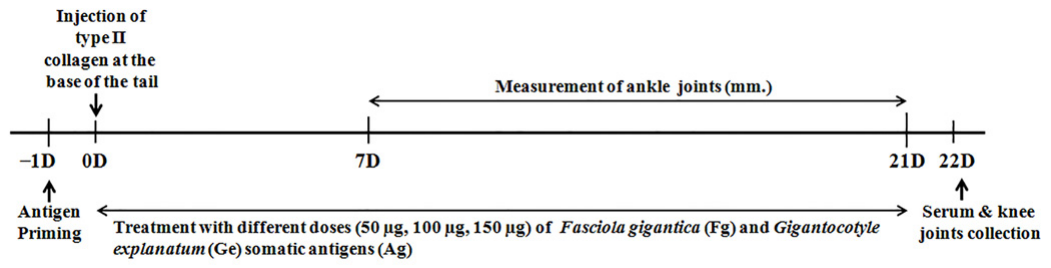
Schematic diagram for treatment schedule. The figure showing timing and duration of arthritis induction and treatment of collagen (chicken type II) inoculated Wistar rats.

The collagen-induced arthritis (CIA) was developed according to the previously described protocols [40] with some modifications. Briefly, female Wistar rats were intradermally immunized at the tail base avoiding the tail vain with 100 μg of chicken type II collagen (generous gift from Prof. Haqqi, Cleveland, U.S.A.) prepared at a concentration of 2mg/ml in 0.05M acetic acid, emulsified with an equal volume of Freund’s Complete Adjuvant (FCA) (Sigma, U.S.A.). Following CIA induction, three different doses (50 μg, 100 μg and 150 μg) of Fg-Ag and Ge-Ag were daily injected subcutaneously at the tail base up to 21days in each rat from treatment groups. The rats in the treatment groups were primed with antigens one day (Day-1) before injection of collagen type II (Day 0), while the control group received only vehicle (PBS). On day 0 the rats were also injected with antigens 6 hours after the collagen injection. Finally, on day 22 the rats were anesthetized by anesthetic ether and immediately afterwards maximum amount of blood was collected by cardiac puncture for serum collection. Thereafter, patellae and knee joint tissues were collected for various studies. For better understanding, the treatment schedule is shown by the schematic diagram (Fig 1).

### Paw thickness measurement

The paw thickness was measured using digital vernier caliper (Digital Caliper, India) by taking ankle joints of both the hind paws. The two independent observers having no knowledge of experimentation accomplished this task and pooled data was analyzed and subjected to statistical analysis.

### Quantitation of serum cytokines

The level of serum anti-inflammatory cytokines, IL-4 and IL-10 and pro-inflammatory cytokines, TNF-α and IFN-γ of the control, CIA and Fg-Ag / Ge-Ag treated rats were analyzed according to the instructions provided with the CBA kit (BD Bioscience, U.S.A.).The data was acquired on LSR II Flow Cytometer at BD FACS Academy, JHCU, New Delhi, India and analyzed using FCAP ARRAY software version 3.0 (BD Bioscience, U.S.A.).

### Preparation of cell free suspension of knee joints

The knee joints were macerated using a mortar and pestle, in 50mM tris- HCl buffer (Sigma, U.S.A.), pH-7.6 and 0.1% Triton X-100 (Sigma, U.S.A.) and then homogenized in Teflon Tissue Homogenizer (Yorco, India) while tissue was kept on ice. The crude extract was sonicated with 3 pulses of 10 seconds with a time gap of one minute, keeping the samples on ice, using Digital Ultrasonicator (Biogen Scientific, India). Homogenate was centrifuged at 10,000×g for 10 minutes at 4°C. Afterward, supernatant was aliquoted and stored at—80°C until use.

### Zymographic detection of matrix metalloproteinases (MMPs) in knee joints

The substrate gel zymography was performed as previously described [41] with some modifications. The SDS polyacrylamide gels co-polymerized with gelatin (BioRad Cat. # 170–6537, U.S.A.) (1 mg/ml) were used to detect the gelatinolytic activity in the tissue extracts of knee joints. A total of 26 μg of protein from each sample was subjected to electrophoresis at a constant voltage of 100V. After electrophoresis, gels were incubated in 2.5% Triton X-100 (4 x 15 min changes) with gentle shaking and then incubated overnight at 37°C in activation buffer (50mM Tris-HCl, pH 7.4, containing 10mM Calcium chloride and 0.05% Brij 35 (Sigma, U.S.A.). Gels were then fixed and stained with Coommassie Brilliant Blue R 250 (Sigma, U.S.A.) prepared in fixative (methanol: glacial acetic acid: water:: 45:10:45) and images were acquired on Gel Doc XR+ (BioRad, U.S.A.). The pixel density / unit volume for each band of different active MMPs and ratio between pro / active MMPs for different experimental groups were calculated by using Image Lab software (BioRad, U.S.A.). The identical gels were incubated in activation buffer containing 20mM EDTA (Sigma, U.S.A.), for MMPs inhibition study.

### Nitric Oxide assay in knee joints

Nitric oxide (NO), an important physiological messenger and effector molecule in immunological systems [42], was determined by a previously described method using the Griess Reagent System [43]. Briefly, the cell free suspension of the joint extract was prepared in 0.05M Tris-HCl buffer, pH-7.6 and then 50 μl of experimental sample was added in 96 well ELISA plate in duplicate followed by addition of 100 μl of Griess reagent (0.04g/ml) (Sigma, U.S.A.). Samples were incubated at room temperature for 10 minutes in dark. The reading was taken at 540nm on iMark Elisa Plate Reader (BioRad, U.S.A.). The level of NO (μM/ mg protein) in unknown sample was measured by comparing with standard curve prepared with sodium nitrite (Fisher Scientific, U.S.A.).

### Scanning Electron Microscopy of patellae

The joints of interest i.e. knee joints were dissected out and patellae were carefully removed and fixed in 2.5% gultaraldehyde / paraformalehyde (Sigma, U.S.A.) solution for 24 hours at 4°C. Further sample processing was done at the All India Institute of Medical Sciences (AIIMS) EM Facility, New Delhi, India, following standard protocol. Samples were visualized on Jeol Scanning Electron Microscope, Model, JSM 6510 LV (USIF, AMU, Aligarh, India) and the images were saved.

### Protein estimation

The protein content in extracts of *F*. *gigantica*, *G*. *explanatum* and knee joints was estimated by the dye binding method [44] as modified for smaller volumes [45]. The bovine serum albumin was used as the standard.

### Statistical Analysis

The data was subjected to Tukey HSD (Tukey high significant difference) One way ANOVA test by using R software [R version 3.1.2 (2014-10-31) “Pumpkin Helmet”] for multiple comparison in all the parameters studied. The graphs for different parameters (cytokines, MMPs and NO) were plotted in the form of box plots which show median, quartile and interquartile range using R software. The paw thickness was plotted on Microsoft excel (Window 7.0) and Mean±SEM was calculated. The Spearman rank correlation coefficient (r^2^), for paw thickness measurement (carried out by two independent observers) was calculated by the above statistical software. The level of significance with the p value less than 0.05 was considered as statistically significant.

## Results

### Induction of Arthritis

**Fig 2:**
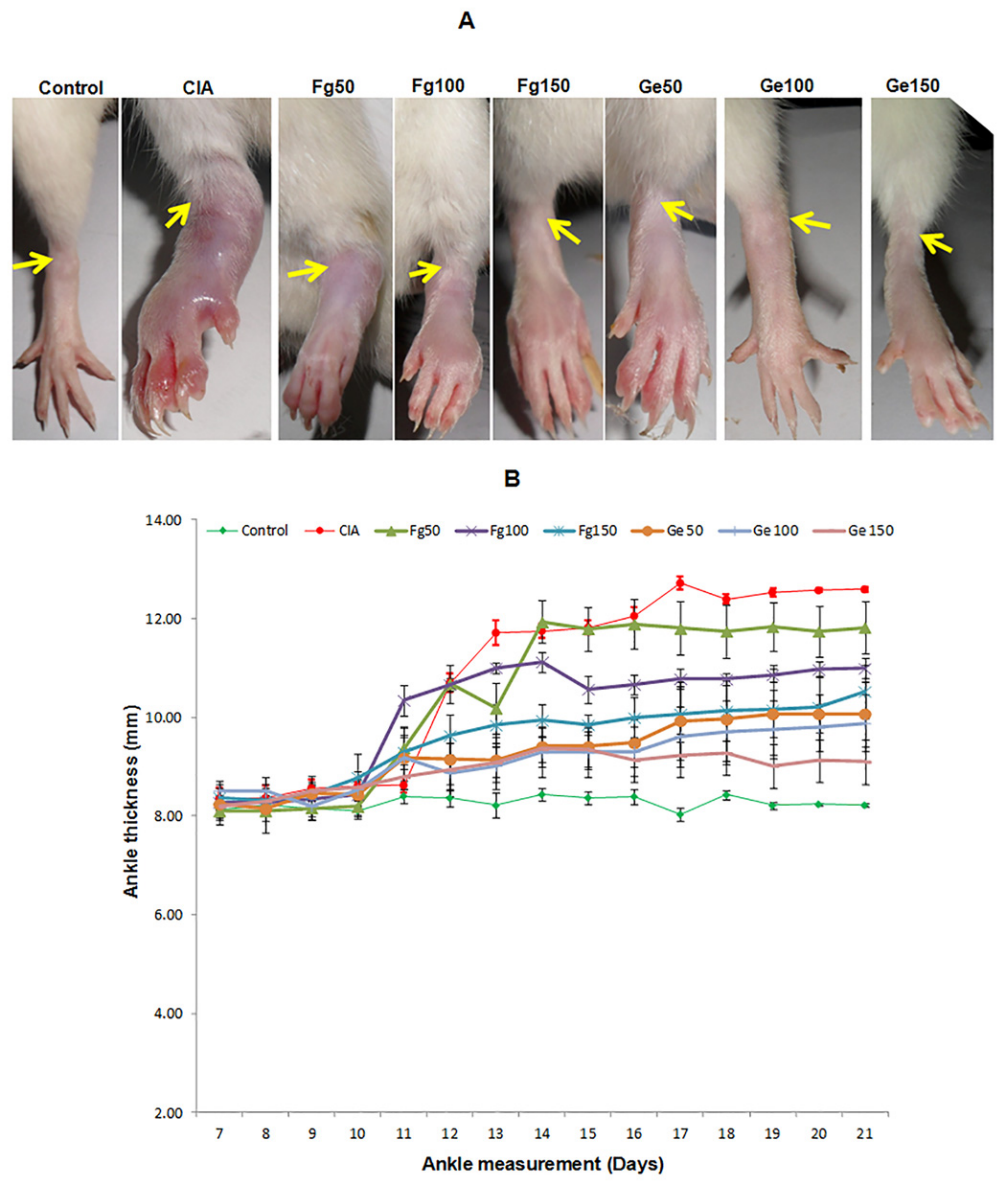
Change in paw thickness of collagen-induced arthritic rats. The results showing disease severity in terms of hind paw thickness as evident in the representative photographs (A) and the plot (B) of the control (vehicle / PBS), collagen-induced arthritis (CIA) and after treatment with different doses, viz. 50 μg, 100 μg and 150 μg, of *F*. *gigantica* and *G*. *explanatum* somatic antigens. The therapeutic significance level of different antigens treated rats in comparison to CIA rats was: Fg50 (p< 0.05), Fg100 (p<0.05), Fg150 (p< 0.01), Ge50 (p< 0.01), Ge100 (p< 0.001), Ge150 (p< 0.001). The values are represented as Mean ± SEM. The p value < 0.05 was considered as significant (A & B). (The data of all the antigens treated groups is compared with the CIA group)

Arthritis was developed promptly in rats inoculated with collagen emulsified with FCA. Clinical emblems of the disease were erythema of one or more ankle joints, followed by metatarsal and interphalangeal joints, first appeared in the hind paws between 8 and 9 days after injecting chicken type II collagen, with a 100% incidence by day 13±1. Swelling, redness and restriction in movement was also observed in CIA rats as compared to the controls (Fig 2A).

### Clinical symptoms of arthritis were modified by liver fluke somatic antigens

The collagen-induced arthritic joints in Wistar rats showed remission of the disease following immunization with Fg-Ag and Ge-Ag. The Ge-Ag was found to be more effective than the Fg-Ag (Fig 2B) but the significant dose dependent response was not evident. The maximum disease suppression occurred at 150 μg of Ge-Ag treatment. From the very beginning the treated rats (except Fg50) consistently showed low to moderate symptoms (redness, swelling and restriction in movement) as compared to the CIA rats. In Fg50 treated rats, the symptoms lowered down significantly after 16^th^ day. The Spearman rank correlation coefficient (r^2^) between data (paw thickness) recorded by two independent observers was: Control (0.932), CIA (0.981), Fg50 (0.938), Fg100 (0.987), Fg150 (0.986), Ge50 (0.989), Ge100 (0.994) and GF150 (0.996). The therapeutic significance level of different antigen treated rats in comparison to CIA rats was: Fg50 (p< 0.05), Fg100 (p< 0.05), Fg150 (p< 0.01), Ge50 (p< 0.01), Ge100 (p< 0.001), Ge150 (p< 0.001) and the order of disease remission was: Ge150 > Ge100 > Ge50 > Fg150 > Fg100 > Fg50.

### Modulation of serum cytokines

**Fig 3:**
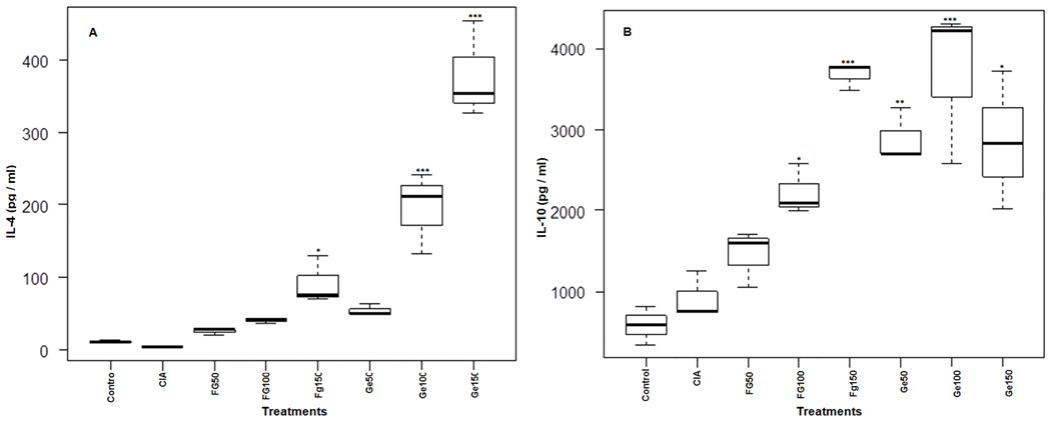
Box plot showing the level of anti-inflammatory serum cytokines (IL-4 and IL-10) in Wistar rats. The level of IL-4 (A) and IL-10 (B) was measured in control rats, CIA rats and experimental rats treated with three different doses (doses; 50 μg, 100 μg and 150 μg) of parasite *F*. *gigantica* and *G*. *explanatum* somatic antigens. Fg50, Fg100 and Ge50 did not exert any significant influence on IL-4 level while in case of IL-10 only Fg50 did not cause any significant effect. The graph is showing median, different quartiles and interquartile range. The p value < 0.05 was considered as significant, where *** < 0.001, ** < 0.01, * < 0.05. (The data of all the antigens treated groups is compared with the CIA group).

The disequilibrium in inflammatory cytokines has a central role in the perpetuation of chronic inflammation and tissue damage during progression of RA. The level anti-inflammatory cytokines, IL-4 and IL-10 and pro-inflammatory cytokines, TNF-α and IFN-γ served as the marker for Th2 and Th1 environment in rat’s serum, respectively. Our results showed significant (p<0.05) increase in the level of IL-4 at 150 μg of Fg-Ag and at higher doses (100 μg and 150 μg) of Ge-Ag (p <0.001) (Fig 3A). In case of Fg-Ag treated rats a significant (p< 0.05 and p< 0.001 for 100μg and 150μg doses, respectively) increase in IL-10 was observed in a dose dependent manner, while no significant change was recorded at 50 μg of Fg-Ag. In case of Ge-Ag treated rats, all the antigenic doses (50 μg, 100 μg, 150 μg) showed a significantly (p<0.01, p< 0.001 and p< 0.05, respectively) increased IL-10 level in a dose independent manner with maximum level at 100 μg of antigen (Fig 3B).

**Fig 4:**
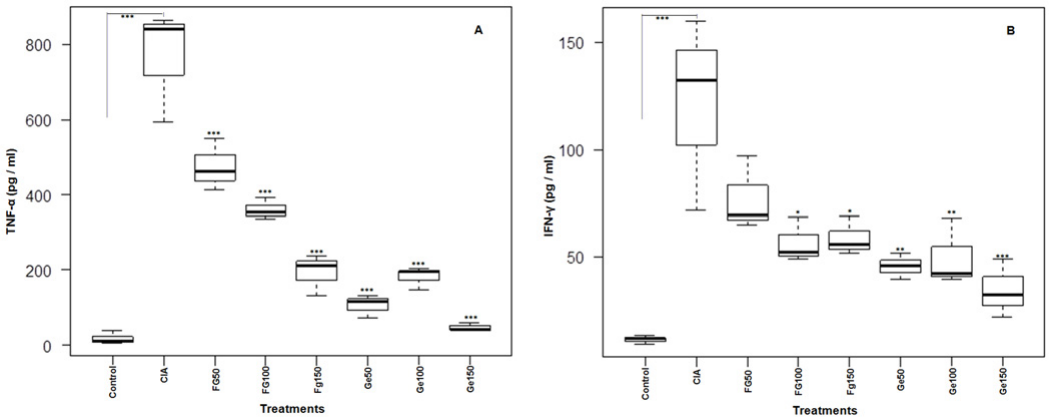
Box plot showing the level of pro-inflammatory serum cytokines (TNF-α and IFN-γ) in Wistar rats. The level of TNF-α (A) and IFN-γ (B) was measured in control, CIA and *F*. *gigantica*, *G*. *explanatum* somatic antigens (doses; 50μg, 100μg and 150μg) treated rats. All the doses of Ge-Ag showed significant depletion of TNF-α and IFN-γ except Fg50 which did not show significant depletion in IFN-γ level. The graph is showing median, different quartiles and interquartile range. The p value <0.05 was considered as significant, where *** < 0.001, ** < 0.01, * < 0.05. (The data of all the antigens treated groups is compared with the CIA group).

A significant (p< 0.001) depletion of TNF-α in serum was observed with dose dependent manner in case of Fg-Ag treated rats, while Ge-Ag treated rats showed a significant (p< 0.001) dose independent depletion in TNF-α level (4A). The level of pro-inflammatory cytokine IFN-γ in CIA rats was significantly (p< 0.001) increased as compared to control rats, but declined significantly (p< 0.05) after treatment with *Fasciola* antigens in a dose independent manner at higher doses (100 μg and 150 μg of Fg-Ag). However, in case of Ge-Ag treated rats IFN-γ level decreased significantly (p<0.01, p< 0.01 and p< 0.001 for 50 μg, 100 μg and 150 μg of Ge-Ag respectively) in a dose dependent manner (Fig 4B).

### Down regulation of MMP-2, MMP-9 and MMP-13

**Fig 5:**
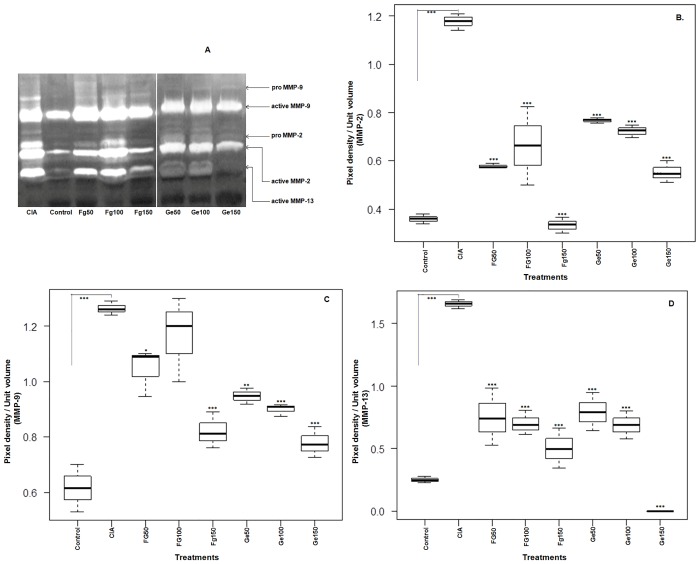
MMPs (Matrix Metalloproteinases) expression in knee joints. The zymogram of matrix metalloproteinases-2, -9 and -13 (MMPs-2, -9 and -13) in joints extracts of control, CIA and rats treated with *F*. *gigantica* and *G*. *explanatum* (Ge) somatic antigens (Ag) (doses; 50 μg, 100 μg and 150 μg) (A). The pixel density (per unit volume) of MMP-2 (B), MMP-9 (C) and MMP-13 (D) was very high in CIA rats as compared to the treated rats, suggesting inhibition of MMPs expression after treatment strategy. The 150 μg of Ge-Ag almost completely wiped out the enzyme activity (D). The graph is showing median, different quartiles and interquartile range. The p value <0.05 was considered as significant, where *** < 0.001, ** < 0.01, *** < 0.05. (The data of all the antigens treated groups is compared with the CIA group)

Both the pro- (latent) and active-MMPs were detected in control, inflamed (CIA) as well as treated knee joints extracts (Fig 5A showing representative gel). A very significant (p< 0.001) increase in different MMPs expression was observed in CIA rats in comparison to control rats, showing the severe pathology and degradation of collagen after injecting type II collagen. There was an overall significant (p< 0.001) inhibition of MMP-2 (Fig 5B), MMP-9 (Fig 5C) and MMP-13 (Fig 5D) in joint extracts of Wistar rats after treatment with different doses of Fg-Ag and Ge-Ag somatic antigens in comparison to the untreated CIA rats. It is evident from Fig 5A that the level of MMP-13 was suppressed more significantly than MMP-2 and MMP-9 by the fluke antigens and it vanished completely at the highest dose of Ge-Ag. In control rats pro MMPs (-2, -9 and -13) were not detectable while in rest of the treatment pro MMP-2 and MMP-9 could be measured except pro MMP-13. The ratio of pro and active MMPs is shown in Table 1. The ratio of pro and active MMP- 2 and 9 was maximum (0.337 and 0.242, respectively) in CIA rats while minimum in Ge150 (0.103) and Fg150 (0.075) treated rats. However, the difference between MMP-2 and MMP-9 ratio was not significant. The gels which were incubated in the activation buffer containing EDTA did not show any gelatinolytic activity, hence confirming the presence of MMPs (S1 Fig).

**Table 1:** Ratio of pro and active MMPs (-2 and -9).

MMPs	Control	CIA	Fg50	Fg100	Fg150	Ge50	Ge100	Ge150
**MMP-2**	0.00	0.337	0.206	0.178	0.154	0.265	0.144	0.103
**MMP-9**	0.00	0.242	0.083	0.110	0.075	0.130	0.142	0.141

### Inhibition of Nitric oxide in joints

**Fig 6:**
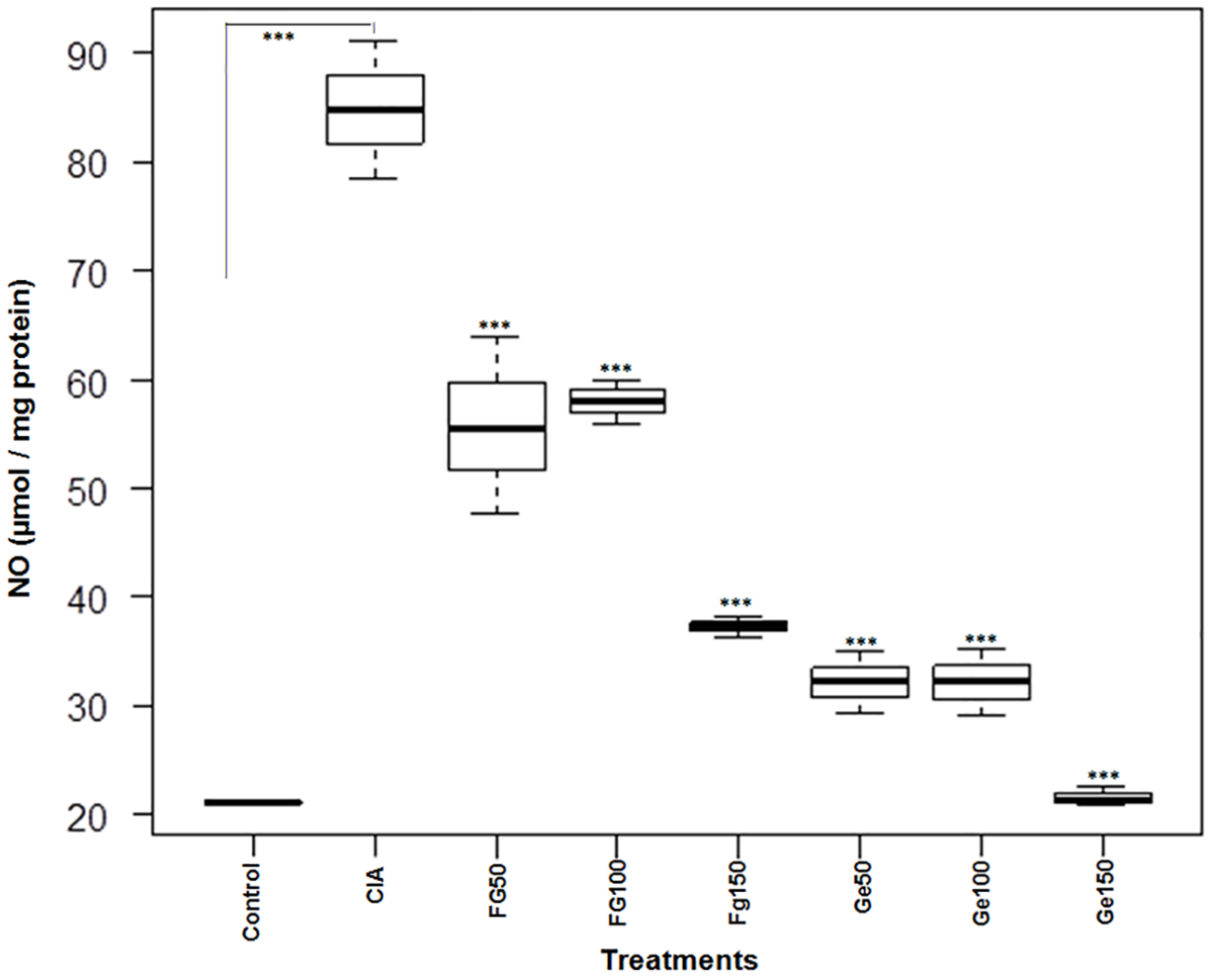
Nitric Oxide (NO) analysis in knee joints. The NO level in joints of control rats, CIA rats and *F*. *gigantica* and *G*. *explanatum* somatic antigens (doses; 50 μg, 100 μg and 150 μg) treated Wistar rats. The graph is showing median, different quartiles and interquartile range. The p value < 0.05 was considered as significant, where *** < 0.001. (The data of all the antigen treated groups is compared with the CIA group).

The NO level was sharply upregulated in CIA rats while treatment of diseased rats with parasite antigens (Fg-Ag and Ge-Ag) significantly (p < 0.001) brought down the level of NO. The effect of Ge-Ag appeared to be more pronounced, particularly at the highest dose (150 μg), which maintained NO level close to the control, reflecting dose dependent inhibitory effect of the Ge-Ag (Fig 6).

### Patellar morphology and interaction with polymorphonuclear cells

**Fig 7:**
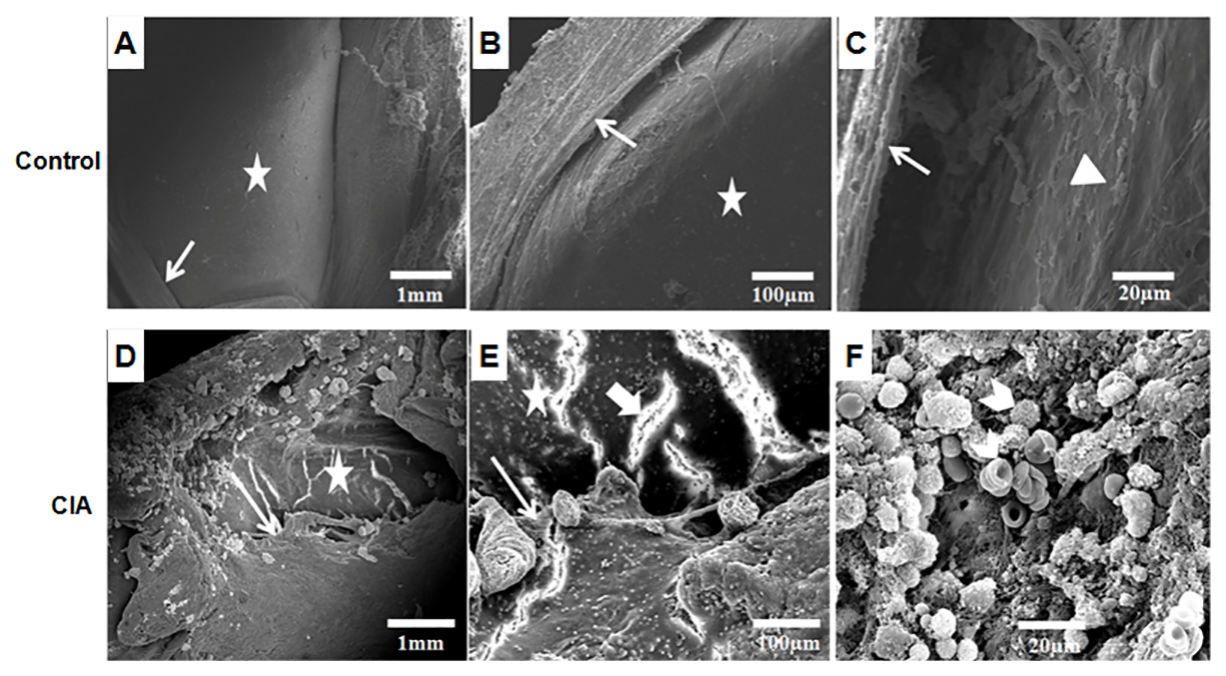
Scanning Electron Microscopy of patellae of healthy and diseased rats. Patellar morphology of control (A-C) and CIA (D-E) Wistar rats. The surface morphology was determined on the basis of fissuring at articular cartilage (wide arrow), synovial hypertrophy (arrow), infiltration of polymorphonuclear cells, vascularization (arrow head), articular cartilage (star) and mucilaginous layer at synovium-cartilage junction (triangle) can be clearly seen.

The scanning electron microscopy revealed that the patellae of control rats appeared healthy and well orchestrated (Fig 7A–7C). Whereas, the CIA rats showed severe pathology with synovial hypertrophy, protrusion of synovial membrane, recruitment of inflammatory cells and prominent fissuring of articular cartilage. The overall disruption and shrinkage of patellae was clearly evident (Fig 7D–7F).

**Fig 8:**
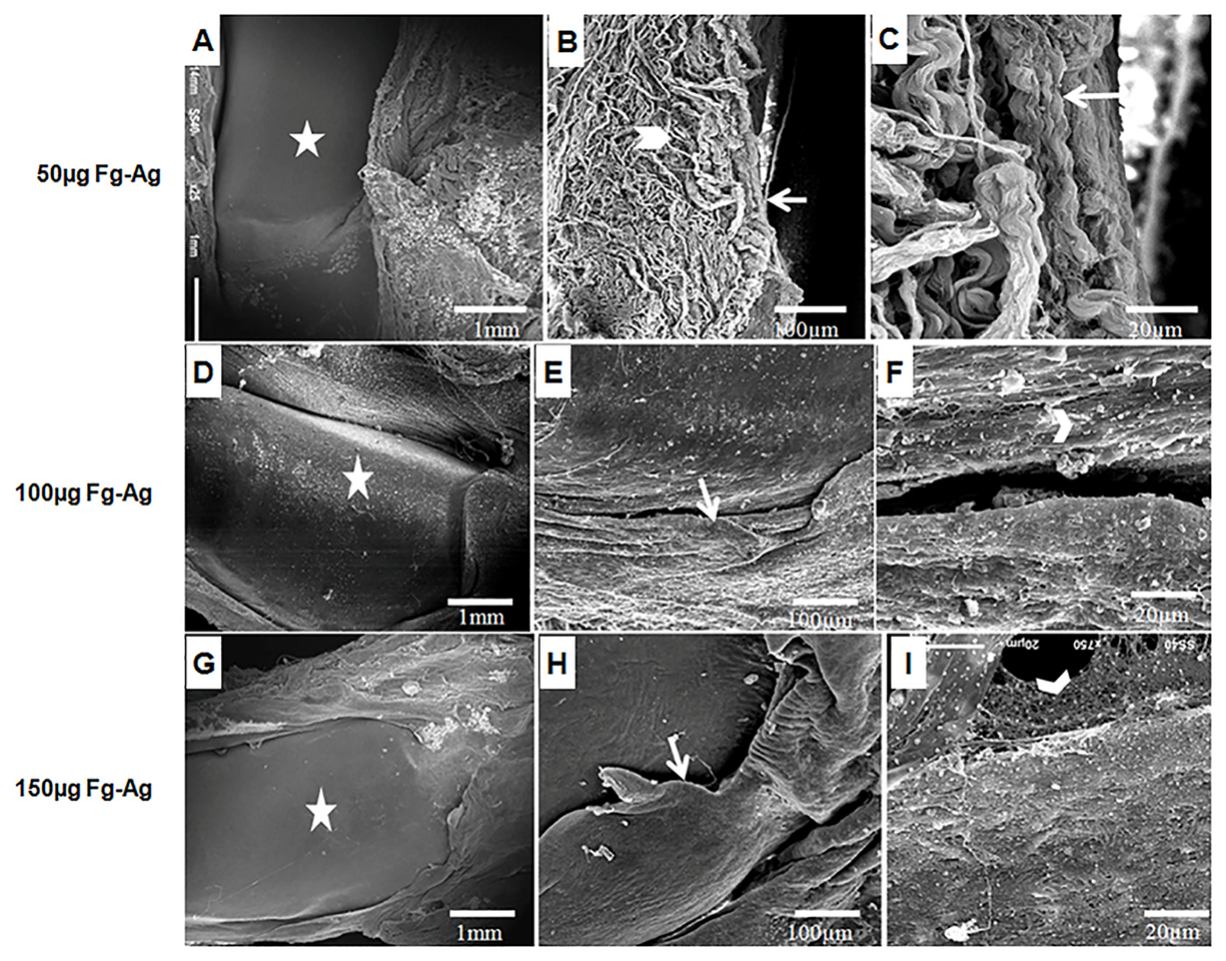
Scanning Electron Microscopy of Patellae of *F*. *gigantica* somatic antigens treated rats. Patellar morphology of Collagen-induced arthritis (CIA) in Wistar rats after treatment with three doses (50 μg, 100 μg and 150 μg) of *Fasciola gigantica* antigens (Fg-Ag) (A-I). Disease remission occurred with limited or no damage to the cartilage (star) (A, D & G). The synovial hypertrophy was prominent (arrow) with loose fibrous meshwork (arrow head) (B, C, E, & F). The protection level increased at the higher doses of the antigen (H & I).

**Fig 9:**
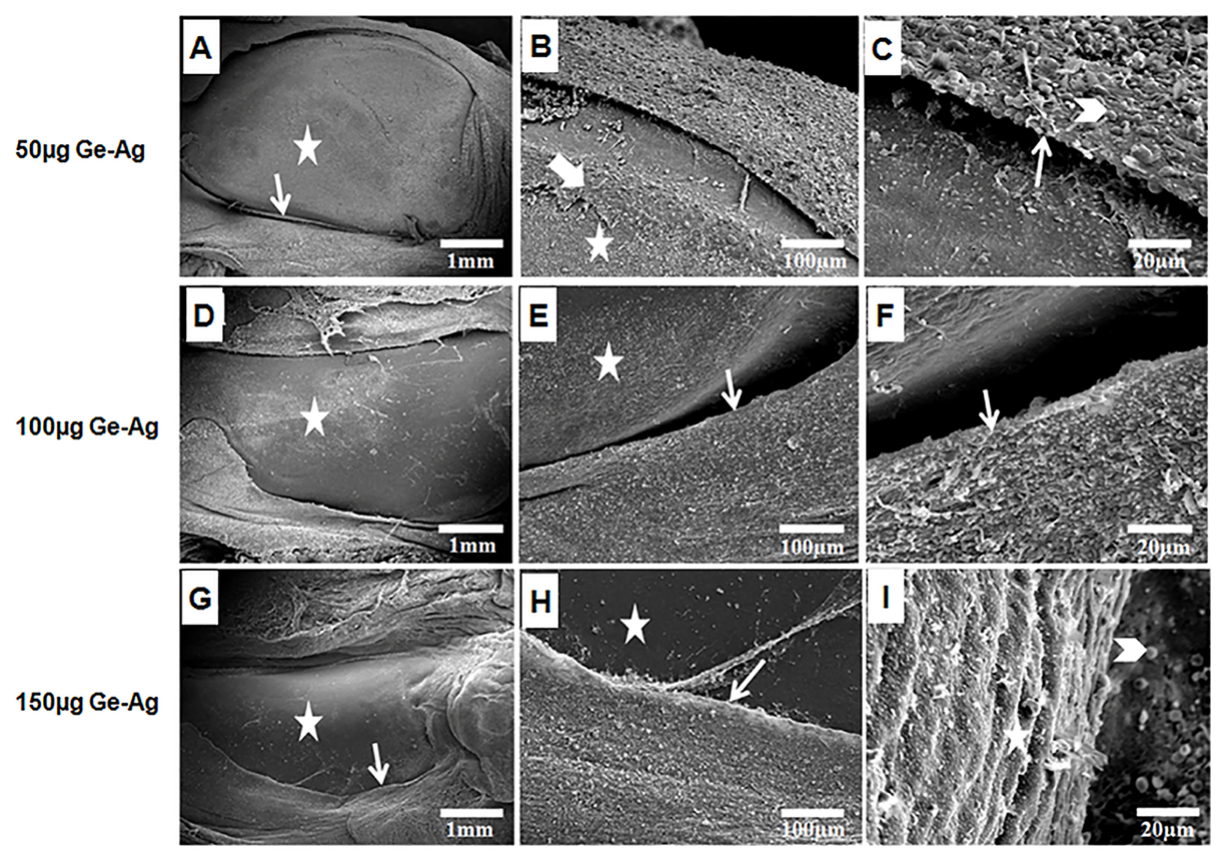
Scanning Electron Microscopy of patellae of *G*. *explanatum* somatic antigens treated rats. Patellar morphology of CIA (Collagen-Induced Arthritis) in Wistar rats after treatment with different doses (50 μg, 100 μg and 150 μg) of *Gigantocotyle explanatum* antigen (Ge-Ag) (A-I). There was a significant disease remission with no cartilage damage (star), no synovial hypertrophy (arrow) but rough surface at synovium-cartilage junction (wide arrow) (B) with intact synovial linings (A-I) and accumulation of some PMNCs were observed (arrow head) (C & I).

Though the patellae in Fg-Ag treated rats was not as healthy as control, but showed some degree of protection with slight synovial hypertrophy and presence of PMNCs at synovium-cartilage junction without any cartilage damage that reflects disease remission (Fig 8A–8I), thus supporting our other results. The patellae of Ge-Ag treated rats showed not only a high degree of disease remission as compared to CIA rats, but also in comparison to Fg-Ag treated rats, thus providing a high level of protection. The highest dose of Ge-Ag (150 μg) resulted in negligible synovial hypertrophy and absence of PMNCs in the joints (Fig 9A–9I).

## Discussion

Experimental infection in mouse models suggests that helminth infection can ameliorate autoimmune diseases [32, 34, 46–50] and provokes a major shift in understanding of T cell regulation. It has been reported that the *Fasciola hepatica* infection as well as its E/S products induce polarized Th2 responses in mice and suppressed Th1 responses [36, 51]. Collagen-induced arthritis is a well known model of autoimmune disorder having many similarities with the rheumatoid arthritis. It is evident from the present results that there was considerable inflammation of joints in the CIA rats along with an increased MMP-2, MMP-9 and MMP-13 activity, which has also been known to be upregulated in humans [52]. The ratio of pro and active MMPs (-2, -9) was maximum in knee joints of diseased rats, while pro MMP-13 was not detectable in diseased as well as in other experimental groups. The difference between the ratio of pro/active MMP-2 and MMP-9 was not significant. In the present study, upregulation of serum TNF-α and IFN-γ level while down regulation of IL-4 and IL-10 level, increased NO level and damages to the patellar morphology was observed in CIA rats. The treatment of arthritic rats with tropical liver fluke, *Fasciola gigantica* and the amphistome, *Gigantocotyle explanatum* derived somatic antigens resulted in significant remission of the disease as manifested by clinical symptoms (swelling, redness and restriction in movement), depletion in serum TNF-α and IFN-γ, increased serum IL-4 and IL-10 levels, under expression of MMPs, decline in NO synthesis and amelioration of patellae pathology. The reason behind this effect could be understood from the epidemiology of autoimmune disorders and helminthic infections that build the background for hygiene hypothesis which proposes that increased incidence of the inflammatory diseases is associated with the absence of appropriate priming of the immune system by parasitic helminths during the childhood [53].

In RA, the disregulation in Th1 immune response leads to the destruction of cartilage and bone through matrix metalloproteinases which disrupt the function of chondrocytes and osteoclasts and high oxidative stress [54, 55]. Furthermore, altered / activated macrophages secrete different cytokines including TNF-α, IL-1 and IL-6, which attract other cells towards the inflammation site [56] where TNF-α along with IL-1 in synovial tissues stimulate release of MMPs, which further enhance the damage [11, 12]. However, the helminth derived somatic products inhibited the Th1 type of immunity in the present study. It has been shown that the nematode secreted product, ES-62, ameliorates the arthritic symptoms [32]. This protective effect can be correlated with the inhibition of collagen specific pro-inflammatory cytokines- TNF-α, IFN-γ, IL-6 and suppression of IgG2a antibody [57]. It has been described earlier that the *Fasciola* infection induces a strong Th2 response leading to the antibody class switching and inhibiting the Th1 response [33]. Studies have also revealed that the *F*. *hepatica* infection can attenuate the experimental autoimmune encephalomyelitis through suppression of Th1 and Th17 immune responses [34]. Furthermore, the parasite derived products also induce strong Th2 response [36, 39]. The analysis of serum cytokines showed that the *Fasciola gigantica* and *Gigantocotyle explanatum* derived somatic antigens also elicited strong Th2 type of immune response in Wistar rats as reflected by high levels of serum IL-4 and IL-10 and low level of TNF-α and IFN-γ in CIA rats after treatment with Fg-Ag and Ge-Ag in the present study. Therefore, the immune suppression involving inhibition of different MMPs (-2, -9 and -13) by the somatic antigens that may also include the excretory / secretory precursors of liver flukes, might be one of the major mechanism that could be protecting the articular cartilage in experimentally induced auto-immune condition. The inhibition of MMP-13 was extraordinarily high in case of *G*. *explanataum* treated rats that may suggest the specificity of inhibition for MMP-13 by this parasite. Such specificity of inhibition may become a major shift in arthritis treatment, because the collagen degradation is mainly initiated by MMP-13 [13, 14]. The explanation of the rationale for such specific inhibition is not clear at this stage but it is well established that different MMPs can be selectively and non-selectively inhibited by different products [18, 58–60]. Therefore, it can be speculated that the *G*. *explanatum* antigens might be containing some molecules which strongly inhibited MMP-13 in particular and MMP-2 and MMP-9 in general, however, there was no significant difference between the cytokine profile of *G*. *explanatum* and *F*. *gigantica* treated rats. Furthermore, high level of NO is the outcome of the disease severity [55] which in turn stimulates the pro inflammatory environment and is an important mediator in central and peripheral pain; promote cartilage damage by modulating MMPs expression and apoptosis [61]. In the present study, inhibition of nitric oxide by the liver fluke antigens also suggests that the collagen mediated Th1 response and MMPs expression was suppressed in the Wistar rats. The destruction of articular cartilage, synovium hypertrophy, recruitment of PMNCs in patellae in CIA rats as evident from the S E M (Scanning Electron Microscopy) results reflect pro-inflammatory environment which was down regulated after treatment with different doses of Fg-Ag and Ge-Ag antigens. Despite of insignificant variation in IL-4, IL-10, TNF-α and IFN-γ level between Fg-Ag and Ge-Ag treated rats, the overall protection level was higher in rats treated with Ge-Ag, which could possibly be due to the interspecific molecular differences that remain a subject for further investigations.

## Conclusion

Taken together, it is concluded that not only the tropical liver fluke, *Fasciola gigantica* but less known amphistome parasite, *Gigantocotyle explanatum* also ameliorates the experimentally induced arthritic symptoms by strong polarization of the immune response towards the Th2 type. However, further studies are required to identify and characterize the active components of the two parasites to validate their anti-inflammatory potential by suppressing the MMPs and NO level as well as the signal transduction pathways which involve T cell polarization.

## Supporting Information

S1 Fig Matrix metalloproteinases (MMPs) inhibition study. Representative zymographic gel incubated in activation buffer containing EDTA.
(TIF)

S2 Fig Schematic diagram showing summary of the work. MMPs; matrix metalloproteinases, IL-10; Interleukin-10, IFN- γ; Interferon-γ, CIA; Collagen induced arthritis, NO; nitric oxide
(TIF)
