# Supplementary figures and images for: Somatic Antigens of Tropical Liver Flukes Ameliorate Collagen-Induced Arthritis in Wistar Rats
Source: PLoS One. 2015 May 18;10(5):e0126429. doi: 10.1371/journal.pone.0126429 (PMC4436316; doi:10.1371/journal.pone.0126429)

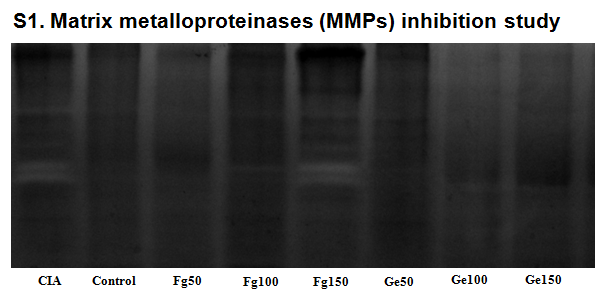

Supplement: S1 Fig — Representative zymographic gel incubated in activation buffer containing EDTA. (TIF) [file pone.0126429.s001.tif]

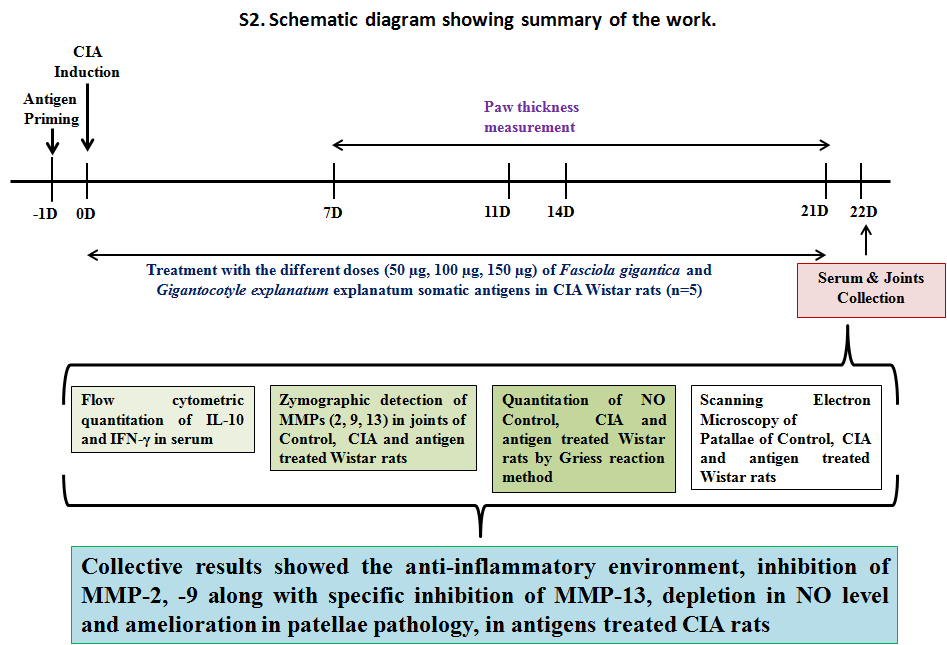

Supplement: S2 Fig — MMPs; matrix metalloproteinases, IL-10; Interleukin-10, IFN- γ; Interferon-γ, CIA; Collagen induced arthritis, NO; nitric oxide (TIF) [file pone.0126429.s002.tif]
